# Phylogenetically Informative Mutations in Drug Resistance Genes of Human-Infecting *Mycobacterium bovis*

**DOI:** 10.1155/2024/5578214

**Published:** 2024-09-26

**Authors:** Yuhui Dong, Xichao Ou, Bing Zhao, Yuanzhi Wang, Yiduo Liu, Ziyi Liu, Haoran Wang, Xin Ge, Yue Nan, Yanlin Zhao, Xiangmei Zhou

**Affiliations:** ^1^ National Key Laboratory of Veterinary Public Health and Safety College of Veterinary Medicine China Agricultural University, Beijing 100193, China; ^2^ National Key Laboratory of Intelligent Tracking and Forecasting for Infectious Diseases National Center for Tuberculosis Control and Prevention Chinese Center for Disease Control and Prevention, Beijing 102206, China

**Keywords:** drug resistance genes, *Mycobacterium bovis*, phylogenetics, tuberculosis and other mycobacterial infections, zoonoses

## Abstract

The diagnosis of drug-resistant tuberculosis (TB) by molecular testing of *Mycobacterium tuberculosis* drug resistance genes is becoming increasingly common clinically. However, *M. bovis*, as an uncommon pathogen of human TB, may interfere with the test results. A comprehensive understanding of phylogenetically informative mutations in the drug resistance genes of *M. bovis* is required to distinguish true resistance-conferring mutations. We analyzed 53 drug resistance genes in 165 *M. bovis* isolated from humans using whole-genome sequencing data and found that 98.2% (162/165) of isolates have pyrazinamide intrinsic genotypic resistance, owing to the H57D mutation in the *pncA* gene. 12.1% (20/165) of *M. bovis* isolates were resistant to drugs other than pyrazinamide. Furthermore, we discovered 18 phylogenetically informative mutations that differed between *M. bovis* and the major lineages 1–4 of *M. tuberculosis*. Additionally, we reported false-positive ethambutol resistance caused by *M. bovis* infection due to the phylogenetically informative mutation *embB* E378A. This study is crucial for gaining insights into the genetic characterization and drug resistance of *M. bovis* prevalent in humans, as well as contributing to the development of more accurate molecular diagnostic methods and detection tools for drug resistance.

## 1. Introduction

Tuberculosis (TB) remains a global health challenge, with the emergence of drug-resistant isolates leading to unfavorable clinical outcomes. The etiological agents of TB are a group of closely related (> 99% nucleotide sequence identity) bacteria known as the *Mycobacterium tuberculosis* complex (MTBC) [1]. The MTBC achieves genetic diversity through genetic mutations, recombination, and natural selection, enabling its widespread global propagation [2]. Genetic mutations within the MTBC primarily drive drug resistance, impacting drug sensitivity testing (DST) and clinical drug use [3]. Certain strains of MTBC exhibit intrinsic resistance to specific drugs due to genetic alterations [4–6]. Owing to the strictly clonal population structure of MTBC and the absence of horizontal gene transfer, these mutations are often unique and phylogenetically informative, serving as markers for specific subgroups within the global MTBC diversity [6]. For instance, the phylogenetic marker *tlyA* N236K of a cluster of *M. tuberculosis* lineage 4.6.2 leads to intrinsic capreomycin (CPM) resistance [5]. H57D in the *pncA* gene of *M. bovis* results in intrinsic resistance to pyrazinamide (PZA) [4].

*Mycobacterium bovis*, a member of the MTBC with a broad range of host species as well as the etiological agent of bovine TB, can be transmitted to humans through the consumption of contaminated dairy products or meat [7–9]. Compared with *M. tuberculosis*, TB caused by *M. bovis* is relatively less common in humans [7, 9]. However, due to the similarities in symptoms and clinical manifestations with *M. tuberculosis* infection, the lack of immediate diagnostic tools to accurately differentiate between the two, and the limited systematic monitoring of *M. bovis* as the cause of TB (especially in underdeveloped countries), the incidence of human *M. bovis* infection is underestimated, and it constitutes a potential serious public health threat [10].

Drug-resistant TB threatens global public health security, and although most human cases of drug-resistant TB are caused by *M. tuberculosis*, infections of humans with drug-resistant *M. bovis* have been reported [11–14]. Due to its broad host range, drug-resistant *M. bovis* has been identified in cattle, sheep, dogs, and other domesticated animals [14–17]. Whole-genome sequencing (WGS) can rapidly and accurately obtain genomic information about isolates, reveal the genetic variation of drug-resistant *M. bovis*, and play an important role in tracing the transmission pathway of *M. bovis* [15, 18, 19]. WGS can provide an important tool and data support for studying the transmission, genetic diversity, and evolution of drug-resistant *M. bovis* among hosts of different species.

In this study, we utilized the WGS data of 165 *M. bovis* isolated from humans to examine 53 genes associated with resistance against 19 drugs. These genes were selected based on their documented roles in drug resistance [3, 6]. Resistance-associated genes with no observed mutations were excluded from the analysis. This analysis aims to enhance our understanding of the genetic traits and drug resistance of *M. bovis* in human infections and to aid in the development of more precise diagnostic methods and detection tools.

## 2. Materials and Methods

### 2.1. Strain Collection

We analyzed the genomes of 165 *M. bovis* isolated from humans, of which 160 were downloaded from SRA, NCBI (http://www.ncbi.nlm.nih.gov/sra/, downloaded on 27 November 2023). Among these, 68 genomes were sourced from Zwyer et al. [20] who collected livestock-associated MTBC genomes and defined phylogenetic lineages. This dataset was supplemented with 17 genomes from the TB clinic of the Tijuana General Hospital in Baja, California, USA, and 75 genomes from publicly available WGS datasets [21] (Supporting Information S1). By analyzing the presence or absence of the RD1 and RD4 genomic regions, we confirmed that all genomes were *M. bovis* rather than BCG or other MTBC species, as described previously [22]. In addition, five *M. bovis* genomes were obtained from the Chinese Center for Disease Control and Prevention, specifically four from Xinjiang and one from Jiangsu. One of the isolates from Xinjiang was determined to be ethambutol (EMB) resistant by a high-resolution melting (HRM) assay [23]. Furthermore, we included 117 *M. tuberculosis* isolates from lineages 1–4 in our genomic analysis to assess the phylogenetic informativeness of the identified mutations (Supporting Information S1).

### 2.2. Whole-Genome Sequence Analysis

The FASTQ files containing the raw sequencing data for all the isolate genomes examined can be obtained from SRA, NCBI, and additional information can be found in Supporting Information S1. Fastp v0.23.4 was used to filter out low-quality bases (Phred score < 20) and residual Illumina adapter contaminations in the FASTQ files [24]. Clean reads from each *M. bovis* genome were analyzed with the Snippy 4.6.0 pipeline [25]. First, reads were mapped to the *M. tuberculosis* H37Rv genome (GenBank ID: NC_000962.3) with BWA v0.7.17 [26]. The variant calling was done by Freebayes v1.3.6, and the vcf files were further annotated using SnpEff v5.0 [27, 28]. A comparative circular genome visualization was mapped using Proksee [29].

### 2.3. Phylogenetic Analysis

Single-nucleotide polymorphisms (SNPs) located in PE/PPE gene families were excluded for the phylogenetic reconstruction due to the difficulties in reliably aligning sequences to the high GC repetitive regions [30, 31, and 32]. We then estimated a maximum likelihood tree using IQ-TREE v2.2.0 [33]. The best-fitting substitution model was selected automatically using the ModelFinder program implemented in IQ-TREE [34]. The phylogenetic analysis was performed with 1000 ultrafast bootstrap replicates. The TVM + F, determined as the best-fit model of nucleotide substitution based on the BIC values reported by the ModelFinder program, was employed [34].

### 2.4. Genotypic Drug Resistance Analysis

For each genome, genotypic drug resistance was predicted using TB-Profiler v4.4.0 [35], covering rifampicin (RIF), isoniazid (INH), EMB, PZA, streptomycin (STM), fluoroquinolones (FQ, including moxifloxacin, ofloxacin, levofloxacin, and ciprofloxacin), amikacin (AMK), CPM, kanamycin (KAN), cycloserine (CYC), ethionamide (ETH), clofazimine (CFZ), para-aminosalicylic acid (PAS), delamanid (DLM), bedaquiline (BDQ), and linezolid (LZD).

## 3. Results

### 3.1. Phylogeny and Genotypic Drug Resistance

**Figure 1:**
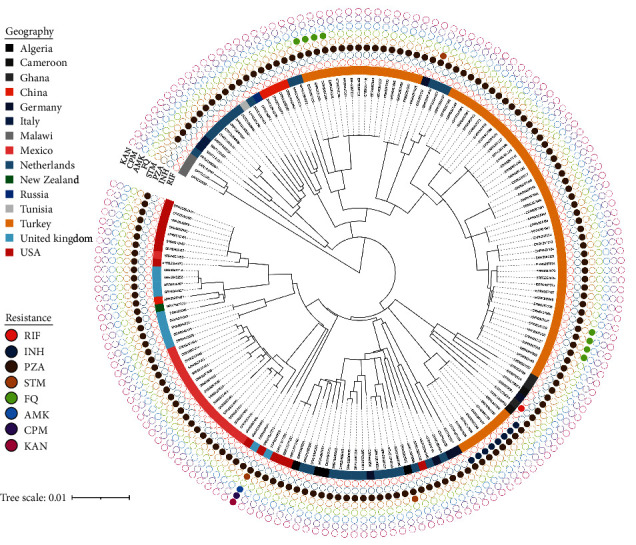
Phylogenetic analysis of 165 *M. bovis* isolated from humans. Maximum-likelihood phylogeny based on 3297 SNPs, with isolates color-coded by geographical origin (inner circle). The resistance profile (outer circle) consists of solid shapes representing the presence of resistance mutations to the drug in the genome of *M. bovis* isolates.

Our collection featured 165 virulent *M. bovis* genomes isolated from humans, originating from Algeria (*n* = 4), Cameroon (*n* = 2), Germany (*n* = 6), Ghana (*n* = 3), Italy (*n* = 2), Malawi (*n* = 3), Mexico (*n* = 17), the Netherlands (*n* = 27), New Zealand (*n* = 1), Russia (*n* = 1), Tunisia (*n* = 1), Turkey (*n* = 68), the United Kingdom (*n* = 11), and the United States of America (*n* = 14), as well as five newly sequenced genomes from China. To better understand the genetic structures of these *M. bovis* genomes, a maximum likelihood tree was constructed based on 3297 SNPs. We used TB-Profiler v4.4.0 to predict the genotypic drug resistance of each *M. bovis* isolate [35]. Overall, 98.2% (162/165) of isolates were resistant to PZA, owing to the H57D mutation in the *pncA* gene. 12.1% (20/165) of *M. bovis* isolates were resistant to drugs other than PZA. Among the 68 isolates in Turkey, in addition to intrinsic resistance to PZA, eight isolates were resistant to INH due to the P2S mutation in the *ahpC* gene, and another eight isolates were resistant to FQ due to the D94G mutation in the *gyrA* gene. An isolate originating from Algeria was resistant to STM due to the *rrs* 888G> A mutation, while two isolates from the Netherlands and the USA were resistant to STM due to the *rpsL* K43R mutation. It is worth noting that this isolate in the USA was also resistant to aminoglycosides (KAN, CPM, and AMK) simultaneously due to the *rrs* 1401A> G mutation. Moreover, a *M. bovis* isolated from Ghana was resistant to RIF due to the *rpoB* Q432R mutation (Figure 1).

### 3.2. Phylogenetically Informative Mutations

**Figure 2:**
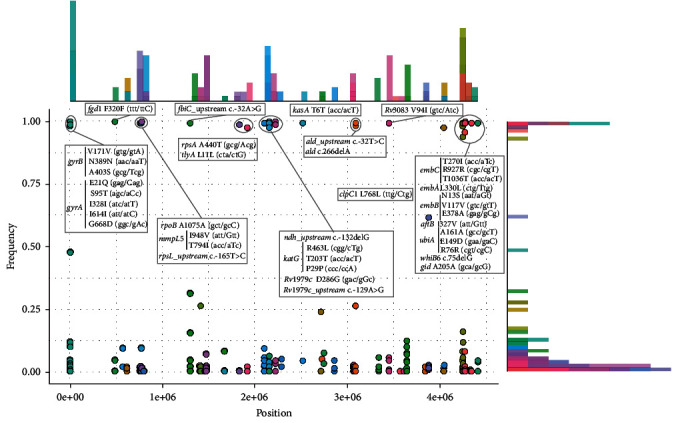
Frequency of mutations in drug resistant genes of *M. bovis* isolated from humans. Forty phylogenetically informative mutations are highlighted by circles. *Rv1979c* is equivalent to *Mb2001c*, and *Rv3083* is equivalent to *Mb3110*.

The *pncA* H57D mutation confers intrinsic PZA resistance to *M. bovis* and serves as a phylogenetically informative mutation [3, 4, 36]. Additionally, we analyzed the mutation frequencies of 53 drug-resistant genes to reveal the phylogenetic polymorphisms that are specific to *M. bovis* but are not associated with drug resistance (Figure 2 and Supporting Information S2). Among the 294 mutations that occurred in these 53 genes, 43.5% (128/294) were low-frequency variations (occurred in only one isolate), compared with 44.9% of SNVs occurring in one isolate of *M. bovis* isolated from cattle, suggesting that *M. bovis* exhibits similar genetic diversity across hosts [15]. Furthermore, we discovered 40 variations with mutation frequencies exceeding 90% (Supporting Information S2). To identify phylogenetically informative markers specific to *M. bovis*, we included 117 genomes from the major lineages 1–4 of *M. tuberculosis* to analyze whether the 40 mutations are also present in these lineages. We found that 18 of these mutations were unique to *M. bovis* and absent in *M. tuberculosis* lineages 1–4 (Table 1). Among them, we identified four mutations that had not previously been recognized as phylogenetically informative markers for *M. bovis*: *clpC1* L768L, *aftB* I327V, *ndh-1*32delG, and *ald* 266delA [6]. The remaining 22 mutations were also present in some *M. tuberculosis* lineages.

### 3.3. Impact of Phylogenetic Polymorphisms on DSTs

**Figure 3:**
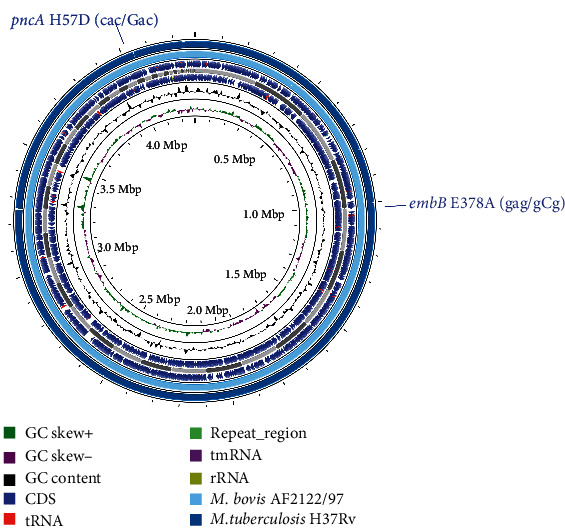
The genome of a *M. bovis* isolate carrying the *embB* E378A mutation. Bacterial genome wide comparisons confirmed a strain of *M. bovis* carrying the *pncA* H57D and *embB* E378A mutations. From inside to outside: ring 1: GC Skew, ring 2: GC content, ring 3: genome annotation, ring 4: BLAST comparison with *M. bovis* AF2122/97, and ring 5: BLAST comparison with *M. tuberculosis* H37Rv. The figure was generated by Proksee (https://proksee.ca/).

The HRM assay is a rapid and simple PCR-based method for detecting SNPs by measuring fluorescence changes in the melting temperature of amplified products [37]. This assay is commonly used for screening drug resistance in *M. tuberculosis* [23, 38]. However, ignoring phylogenetic polymorphisms may lead to false-positive reports of drug resistance [39]. In one case from Xinjiang described in this study, the hospital used a HRM assay to test EMB drug sensitivity in an isolate before confirming it as *M. bovis*. This assay covers the detection of mutations in six codons (306, 368, 378, 380, 406, and 497) located on the *embB* gene [23]. The determination of EMB resistance was made by comparing the melting temperature differences in the melting curves between the tested samples and the positive control. Given the determination of EMB resistance, a customized treatment regimen consisting of INH, RIF, PZA, and levofloxacin (LVX) was implemented, extending the treatment duration to 9 months. However, after WGS, we found that this isolate belongs to *M. bovis*. Through whole-genome BLAST comparisons with the reference strains *M. bovis* AF2122/97 and *M. tuberculosis* H37Rv, we identified an H57D mutation in the *pncA* gene and an E378A mutation in the *embB* gene (Figure 3). These two mutations were present in both the isolate and the *M. bovis* AF2122/97 reference strain. The H57D mutation in the *pncA* gene is associated with the inherent PZA resistance of *M. bovis*, while the E378A mutation in the *embB* gene is unrelated to EMB resistance [3, 40].

**Figure 4:**
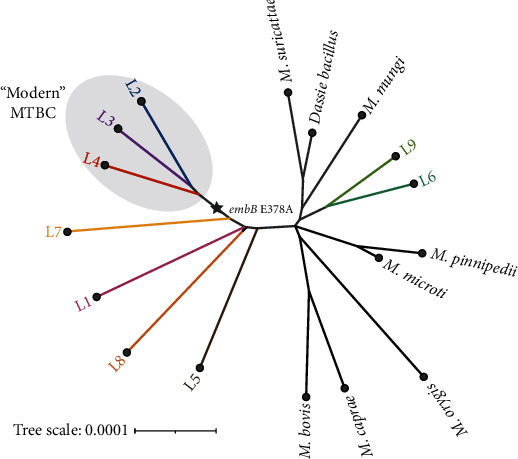
*embB* E378A is a phylogenetically informative marker of MTBC. At this codon, A represents the ancestral amino acid, present in *M. tuberculosis* lineages L1, L5, L6, L7, L8, and L9 and animal-adapted members of the MTBC, whereas E is present in modern MTBC (*M. tuberculosis* L2, L3, and L4 lineages).

Despite emerging from a common progenitor, the MTBC is divided into different phylogenetic lineages. After the loss of the *M. tuberculosis*-specific deletion region 1 (TbD1), *M. tuberculosis* diverged into lineages 2, 3, and 4, known as modern MTBC. In contrast, strains with an intact TbD1 region are referred to as “ancestral” strains [41, 42]. The *embB* E378A mutation is not a marker for EMB resistance but a phylogenetically informative marker of the MTBC [40]. At this codon, A represents the ancestral amino acid, whereas E is present in modern MTBC (Figure 4). Therefore, when the tested isolate belongs to *M. tuberculosis* lineages L1, L5, L6, L7, L8, and L9 or animal-adapted members of the MTBC, using this assay kit can lead to erroneous reports of EMB resistance, impacting drug treatment regimen selection. In this case, due to the false-positive report for EMB resistance, the treatment regimen for the patient excluded EMB and instead used the second-line anti-TB drug LVX, with the treatment duration extended from 6 to 9 months. Following our feedback, the assay used in this case has removed the detection of this mutation. This case highlights the importance of studying phylogenetically informative mutations related to drug resistance genes in *M. bovis*. Ignoring phylogenetic diversity in molecular detection can lead to false-positive results, thereby affecting clinical treatment decisions.

## 4. Discussion

While some countries have made progress in eradicating bovine TB, the disease continues to threaten people who depend on livestock for their livelihoods, particularly in underdeveloped and rural areas. The genome sequence of *M. bovis* is over 99.95% identical to that of *M. tuberculosis*, and many pathogen-associated molecular patterns are identical between the two, allowing recognition by various pattern recognition receptors on macrophages [43, 44]. *M. bovis* can be transmitted to humans through direct contact with infected animals or by consuming animal products such as unpasteurized milk [8]. Though rare, human-to-human transmission of *M. bovis* is also possible [45–47]. As a result, the risk of *M. bovis* infection cannot be entirely eliminated, even in individuals with no prior exposure to farm animals or consumption of pasteurized milk [21, 45, 47]. According to the WHO statistics, *M. bovis* causes ~140,000 new cases of TB and 11,400 deaths in humans each year [48].

Infection with *M. bovis* in humans can result in various symptoms of TB, including cough, poor appetite, fatigue, weight loss, and night sweats [49]. Given that 98.2% (162/165) of *M. bovis* isolated from humans are inherently genotypically resistant to PZA, it is critical to accurately identify *M. bovis* as the cause of human TB before initiating treatment. Although PZA is commonly used in first-line anti-TB treatment, the treatment regimen for *M. bovis* infection is usually adjusted to a combination of RIF, INH, and EMB, with a duration of 9 months [50]. However, due to limitations in PZA resistance testing, cases of *M. bovis* infection may lead to ineffective use of PZA if not accurately diagnosed.

The prevalence of drug-resistant *M. bovis* varies widely between regions. A study showed that of 167 human *M. bovis* infection cases in San Diego, 7% were resistant to INH and 1% to RIF [51]. Another study based on 15 years of laboratory surveillance in Mexico found a high rate of resistance to first-line anti-TB drugs in *M. bovis* isolates from humans, of which 16.1% were resistant to INH, 9.5% to RIF, 0.7% to EMB, 11.8% to STM, and 7.6% were multidrug-resistant [52]. About 70% of isolates were resistant to first- and second-line anti-TB drugs in Peshawar, Pakistan [53]. Our data support the prevalence of drug-resistant *M. bovis* in humans and account for 12.1% of human *M. bovis* infections. The heterogeneity of drug-resistant *M. bovis* prevalence in different regions reflects the genetic diversity and adaptability of *M. bovis*. Ongoing surveillance and testing efforts are key to the timely detection of drug-resistant strains and the adoption of isolation and treatment measures.

Moreover, if the *M. bovis* infection cannot be accurately identified, as illustrated in our case, it can result in false-positive DST, posing a hidden challenge in accurately diagnosing TB and potentially leading to the inability to use the most appropriate treatment scheme. We are confronted with the reality that drug-resistant *M. bovis* is spreading in humans, presenting significant challenges to global public health and clinical treatment [13]. If left unaddressed, this drug resistance could lead to a myriad of adverse outcomes, including reduced cure rates, the need for prolonged treatment, increased overall costs, and potentially treatment failure or even death [54, 55]. Given the gravity of this situation, it is imperative to intensify our efforts in surveillance, prevention, and control measures for zoonotic TB [9]. Early identification of cases of this specific strain of TB is crucial, especially in patients with a history of contact with farm animals or occupational risks. Timely detection and diagnosis of TB cases are essential for identifying the involved strains and implementing appropriate safety measures to prevent further spread [56, 9]. Furthermore, a comprehensive understanding of the epidemiology of drug-resistant *M. bovis* strains is vital for interrupting the transmission chain. This underscores the need for increased emphasis on epidemiological research to gain insights into the disease's spread and its evolution over time. Monitoring resistance genes and utilizing this information to develop effective strategies for combating zoonotic TB is also essential.

Besides the *pncA* H57D mutation being identified as a phylogenetic marker for *M. bovis* and conferring intrinsic resistance to PZA, we also analyzed mutations in 53 genes associated with drug resistance [3, 6]. We discovered 18 phylogenetically informative mutations that can be used to distinguish *M. bovis* from the major lineages 1–4 of *M. tuberculosis*. By recognizing specific phylogenetically informative mutations, we can track the origin and transmission pathways of *M. bovis* more accurately. Additionally, understanding mutations that do not affect drug resistance but have important phylogenetic implications helps us better comprehend the population structure and genetic diversity of *M. bovis*. The most important aspect is to utilize the information obtained from systematic phylogenetic diversity to develop more precise and efficient molecular diagnostic methods, enabling accurate and rapid detection of drug-resistant *M. bovis*, guiding clinical treatment choices, and ultimately achieving effective therapeutic outcomes.

In conclusion, this study revealed the threat of *M. bovis* to human health and the prevalence of drug-resistant *M. bovis* in humans. The association between drug resistance in *M. bovis* and its phylogenetic diversity provides important evidence for accurately tracking the transmission pathways of *M. bovis* and developing precise and efficient molecular diagnostic methods.

## Figures and Tables

**Table 1:** Phylogenetically informative markers distinguishing *M. bovis* from major *M. tuberculosis* lineages 1–4.

Position	Gene name	*M. tuberculosis* H37Rv	*M. bovis* AF2122/97	Variant type	Change
5752	*gyrB*	*Rv0005*	*Mb0005*	synonymous_variant	V171V (gtg/gtA)
6406	*gyrB*	*Rv0006*	*Mb0005*	synonymous_variant	N389N (aac/aaT)
6446	*gyrB*	*Rv0005*	*Mb0005*	missense_variant	A403S (gcg/Tcg)
8285	*gyrA*	*Rv0006*	*Mb0006*	synonymous_variant	I328I (atc/atT)
1302899	*fbiC_upstream*	—	—	upstream_gene_variant	c.-32A > G
1834859	*rpsA*	*Rv1630*	*Mb1656*	missense_variant	A440T (gcg/Acg)
2103173	*ndh_upstream*	—	—	upstream_gene_variant	c.-132delG
2155503	*katG*	*Rv1908c*	*Mb1943c*	synonymous_variant	T203T (acc/acT)
2156025	*katG*	*Rv1908c*	*Mb1943c*	synonymous_variant	P29P (ccc/ccA)
3087084	*ald*	*Rv2780*	*Mb2802*, *Mb2803*	frameshift_variant	c.266delA p.Gln89fs
3448783	*Rv3083*	*Rv3083*	*Mb3110*	missense_variant	V94I (gtc/Atc)
4038403	*clpC1*	*Rv3596c*	*Mb3627c*	synonymous_variant	L768L (ttg/Ctg)
4242970	*embC*	*Rv3794*	*Mb3822*	upstream_gene_variant	T1036T (acc/acT)
4244220	*embA*	*Rv3794*	*Mb3822*	synonymous_variant	L330L (ctg/Ttg)
4246551	*embB*	*Rv3795*	*Mb3824*	missense_variant	N13S (aat/aGt)
4246864	*embB*	*Rv3795*	*Mb3824*	synonymous_variant	V117V (gtc/gtT)
4267858	*aftB*	*Rv3805c*	*Mb3835c*	missense_variant	I327V (att/Gtt)
4269351	*ubiA*	*Rv3806c*	*Mb3836c*	synonymous_variant	A161A (gcc/gcT)

*Note:* Italic value highlights gene names and locus tags.

## Data Availability

The Illumina reads for the five newly sequenced *M. bovis* isolated from China were deposited in the SRA from the NCBI (https://www.ncbi.nlm.nih.gov/) under the project accession number PRJNA937047.

## Supporting Information

Supporting Information S1
*Mycobacterium bovis* and *M. tuberculosis* isolates used in this study.

Supporting Information S2 A catalogue of 40 phylogenetically informative mutations in 53 drug resistance genes.
