# Supplementary material for: Phylogenetically Informative Mutations in Drug Resistance Genes of Human-Infecting Mycobacterium bovis
Source: Transbound Emerg Dis. 2024 Sep 26;2024:5578214. doi: 10.1155/2024/5578214 (PMC12017247; doi:10.1155/2024/5578214)
Supplement: Supporting Information S1 — Mycobacterium bovis and M. tuberculosis isolates used in this study. [file 5578214.f1.pdf]

165 *M. bovis* isolated from humans used in this study		
**SRA ID**	**Country**	**Source**
ERR550755	Germany	Walker et al., 2015
ERR550954	Germany	Walker et al., 2015
ERR551009	Germany	Walker et al., 2015
ERR551252	Germany	Walker et al., 2015
ERR551917	Germany	Walker et al., 2015
ERR553061	Germany	Walker et al., 2015
ERR1203064	Ghana	Otchere et al., 2019
ERR502499	Ghana	Otchere et al., 2019
ERR502526	Ghana	Otchere et al., 2019
SRR7131025	Italy	Cabibbe et al., 2018
SRR7131117	Italy	Cabibbe et al., 2018
ERR161044	Malawi	Brites et al., 2018
ERR181980	Malawi	Brites et al., 2018
ERR212091	Malawi	Brites et al., 2018
SRR5657400	Mexico	Sandoval-Azuara et al., 2017
SRR5657401	Mexico	Sandoval-Azuara et al., 2017
SRR5657408	Mexico	Sandoval-Azuara et al., 2017
SRR5657409	Mexico	Sandoval-Azuara et al., 2017
SRR5657410	Mexico	Sandoval-Azuara et al., 2017
SRR5657464	Mexico	Sandoval-Azuara et al., 2017
SRR5657465	Mexico	Sandoval-Azuara et al., 2017
SRR5657477	Mexico	Sandoval-Azuara et al., 2017
SRR5657478	Mexico	Sandoval-Azuara et al., 2017
SRR5657479	Mexico	Sandoval-Azuara et al., 2017
SRR5657480	Mexico	Sandoval-Azuara et al., 2017
SRR5657481	Mexico	Sandoval-Azuara et al., 2017
SRR5657482	Mexico	Sandoval-Azuara et al., 2017
SRR5657483	Mexico	Sandoval-Azuara et al., 2017
SRR5657484	Mexico	Sandoval-Azuara et al., 2017
SRR5657485	Mexico	Sandoval-Azuara et al., 2017
SRR5657486	Mexico	Sandoval-Azuara et al., 2017
ERR229952	Russia	Casali et al., 2014
SRR3085259	Tunisia	Schena et al., 2016
ERR400386	United Kingdom	Walker et al., 2015
ERR400460	United Kingdom	Walker et al., 2015
SRR6044919	United Kingdom	Quan et al., 2018
SRR6045019	United Kingdom	Quan et al., 2018
SRR6045806	United Kingdom	Quan et al., 2018
SRR6045827	United Kingdom	Quan et al., 2018
SRR6046252	United Kingdom	Quan et al., 2018
SRR6046738	United Kingdom	Quan et al., 2018
SRR6045214	United Kingdom	Quan et al., 2018
SRR6045235	United Kingdom	Quan et al., 2018
SRR6045407	United Kingdom	Quan et al., 2018
SRR4019490	USA	Pattané et al., 2017
SRR7617710	New Zealand	Loiseau et al., 2020
SRR8902557	USA	Shockey et al., 2019
SRR8902559	USA	Shockey et al., 2019
SRR8902560	USA	Shockey et al., 2019
SRR8902561	USA	Shockey et al., 2019
SRR8902562	USA	Shockey et al., 2019
SRR8902563	USA	Shockey et al., 2019
ERR3587501	Algeria	URMITE, France

ERR3587591	Algeria	URMITE, France
ERR3587597	Algeria	URMITE, France
ERR3588222	Algeria	URMITE, France
SRR12554470	USA	USDA, USA
SRR12554471	USA	USDA, USA
ERR6367070	Turkey	Zwyer et al., 2021
ERR6367071	Turkey	Zwyer et al., 2021
ERR6367072	Turkey	Zwyer et al., 2021
ERR6367073	Turkey	Zwyer et al., 2021
ERR6367074	Turkey	Zwyer et al., 2021
ERR6367075	Turkey	Zwyer et al., 2021
ERR6367076	Turkey	Zwyer et al., 2021
ERR6367077	Turkey	Zwyer et al., 2021
ERR6367078	Turkey	Zwyer et al., 2021
ERR6367079	Turkey	Zwyer et al., 2021
ERR6367080	Turkey	Zwyer et al., 2021
ERR6367081	Turkey	Zwyer et al., 2021
ERR6367082	Turkey	Zwyer et al., 2021
ERR6367083	Turkey	Zwyer et al., 2021
ERR6367084	Turkey	Zwyer et al., 2021
ERR6367085	Turkey	Zwyer et al., 2021
ERR6367086	Turkey	Zwyer et al., 2021
ERR6367087	Turkey	Zwyer et al., 2021
ERR6367088	Turkey	Zwyer et al., 2021
ERR6367089	Turkey	Zwyer et al., 2021
ERR6367090	Turkey	Zwyer et al., 2021
ERR6367091	Turkey	Zwyer et al., 2021
ERR6367092	Turkey	Zwyer et al., 2021
ERR6367093	Turkey	Zwyer et al., 2021
ERR6367094	Turkey	Zwyer et al., 2021
ERR6367095	Turkey	Zwyer et al., 2021
ERR6367096	Turkey	Zwyer et al., 2021
ERR6367097	Turkey	Zwyer et al., 2021
ERR6367098	Turkey	Zwyer et al., 2021
ERR6367099	Turkey	Zwyer et al., 2021
ERR6367100	Turkey	Zwyer et al., 2021
ERR6367101	Turkey	Zwyer et al., 2021
ERR6367102	Turkey	Zwyer et al., 2021
ERR6367103	Turkey	Zwyer et al., 2021
ERR6367104	Turkey	Zwyer et al., 2021
ERR6367105	Turkey	Zwyer et al., 2021
ERR6367106	Turkey	Zwyer et al., 2021
ERR6367107	Turkey	Zwyer et al., 2021
ERR6367108	Turkey	Zwyer et al., 2021
ERR6367109	Turkey	Zwyer et al., 2021
ERR6367110	Turkey	Zwyer et al., 2021
ERR6367111	Turkey	Zwyer et al., 2021
ERR6367112	Turkey	Zwyer et al., 2021
ERR6367117	Turkey	Zwyer et al., 2021
ERR6367118	Turkey	Zwyer et al., 2021
ERR6367119	Turkey	Zwyer et al., 2021
ERR6367120	Turkey	Zwyer et al., 2021
ERR6367121	Turkey	Zwyer et al., 2021
ERR6367122	Turkey	Zwyer et al., 2021
ERR6367123	Turkey	Zwyer et al., 2021

ERR6367124	Turkey	Zwyer et al., 2021
ERR6367125	Turkey	Zwyer et al., 2021
ERR6367126	Turkey	Zwyer et al., 2021
ERR6367127	Turkey	Zwyer et al., 2021
ERR6367128	Turkey	Zwyer et al., 2021
ERR6367057	Turkey	Zwyer et al., 2021
ERR6367058	Turkey	Zwyer et al., 2021
ERR6367059	Turkey	Zwyer et al., 2021
ERR6367060	Turkey	Zwyer et al., 2021
ERR6367061	Turkey	Zwyer et al., 2021
ERR6367062	Turkey	Zwyer et al., 2021
ERR6367063	Turkey	Zwyer et al., 2021
ERR6367064	Turkey	Zwyer et al., 2021
ERR6367065	Turkey	Zwyer et al., 2021
ERR6367066	Turkey	Zwyer et al., 2021
ERR6367067	Turkey	Zwyer et al., 2021
ERR6367068	Turkey	Zwyer et al., 2021
ERR6367069	Turkey	Zwyer et al., 2021
SRR17077290	USA	USDA, USA
SRR17077291	USA	USDA, USA
SRR17077298	USA	USDA, USA
SRR21689501	Netherlands	RIVM, Netherlands
SRR21689506	Netherlands	RIVM, Netherlands
SRR21689590	Netherlands	RIVM, Netherlands
SRR21689636	Netherlands	RIVM, Netherlands
SRR21689968	Netherlands	RIVM, Netherlands
SRR21690029	Netherlands	RIVM, Netherlands
SRR21690057	Netherlands	RIVM, Netherlands
SRR21690094	Netherlands	RIVM, Netherlands
SRR21690134	Netherlands	RIVM, Netherlands
SRR21690315	Netherlands	RIVM, Netherlands
SRR21690321	Netherlands	RIVM, Netherlands
SRR21690389	Netherlands	RIVM, Netherlands
SRR21690399	Netherlands	RIVM, Netherlands
SRR21690642	Netherlands	RIVM, Netherlands
SRR21690650	Netherlands	RIVM, Netherlands
SRR21690689	Netherlands	RIVM, Netherlands
SRR21690786	Netherlands	RIVM, Netherlands
SRR21690810	Netherlands	RIVM, Netherlands
SRR21690870	Netherlands	RIVM, Netherlands
SRR21690871	Netherlands	RIVM, Netherlands
SRR21691010	Netherlands	RIVM, Netherlands
SRR21691063	Netherlands	RIVM, Netherlands
SRR21691072	Netherlands	RIVM, Netherlands
SRR21691113	Netherlands	RIVM, Netherlands
SRR21691114	Netherlands	RIVM, Netherlands
SRR21691135	Netherlands	RIVM, Netherlands
SRR21691146	Netherlands	RIVM, Netherlands
SRR23902049	USA	Sundararaman et al., 2023
SRR24827273	USA	Wadsworth Center, USA
ERR11268053	Cameroon	Rossi et al., 2023
ERR11268054	Cameroon	Rossi et al., 2023
SRR23562877	China	This study
SRR23562878	China	This study
SRR23562879	China	This study

SRR23562880	China	This study
SRR23562881	China	This study

117 *M. tuberculosis* isolates used in this study			
**SRA ID**	**Lineage**	**Lineage name**	**Source**
ERR234155	1.1.1	EAI	Comas et al., 2013
SRR022875	1.1.1	EAI	Comas et al., 2013
ERR234156	1.1.1	EAI	Comas et al., 2013
ERR234194	1.1.1	EAI	Comas et al., 2013
ERR233363	1.1.1	EAI	Comas et al., 2013
ERR234207	1.1.1.1	EAI	Comas et al., 2013
ERR234157	1.1.1.1	EAI	Comas et al., 2013
ERR234241	1.1.1.1	EAI	Comas et al., 2013
ERR233365	1.1.1.1	EAI	Comas et al., 2013
ERR234205	1.1.2	EAI	Comas et al., 2013
ERR233351	1.1.2	EAI	Comas et al., 2013
ERR234236	1.1.2	EAI	Comas et al., 2013
ERR233372	1.1.2	EAI	Comas et al., 2013
SRR006916	1.1.3	EAI	Comas et al., 2013
ERR233376	1.1.3	EAI	Comas et al., 2013
SRR017356	1.2.1	EAI Manila	Comas et al., 2013
ERR234262	1.2.1	EAI Manila	Comas et al., 2013
ERR234214	1.2.1	EAI Manila	Comas et al., 2013
ERR234264	1.2.1	EAI Manila	Comas et al., 2013
ERR234266	1.2.1	EAI Manila	Comas et al., 2013
ERR234272	1.2.1	EAI Manila	Comas et al., 2013
ERR234191	1.2.2	EAI	Comas et al., 2013
ERR233377	1.2.2	EAI	Comas et al., 2013
ERR233353	1.2.2	EAI	Comas et al., 2013
ERR233380	1.2.2	EAI	Comas et al., 2013
ERR233356	1.2.2	EAI	Comas et al., 2013
ERR233364	1.2.2	EAI	Comas et al., 2013
ERR234164	1.2.2	EAI	Comas et al., 2013
ERR234248	2.1	East-Asian non-Beijing	Comas et al., 2013
ERR234252	2.1	East-Asian non-Beijing	Comas et al., 2013
ERR234216	2.1	East-Asian non-Beijing	Comas et al., 2013
ERR234135	2.2.1	Beijing	Comas et al., 2013
ERR234253	2.2.1	Beijing	Comas et al., 2013
ERR234098	2.2.1	Beijing	Comas et al., 2013
ERR234124	2.2.1	Beijing	Comas et al., 2013
ERR234132	2.2.1	Beijing	Comas et al., 2013
ERR234118	2.2.1	Beijing	Comas et al., 2013
ERR234123	2.2.1	Beijing	Comas et al., 2013
ERR234119	2.2.1	Beijing	Comas et al., 2013
ERR234193	2.2.1	Beijing	Comas et al., 2013
ERR234245	2.2.1	Beijing	Comas et al., 2013
ERR234126	2.2.1	Beijing	Comas et al., 2013
ERR234270	2.2.1	Beijing	Comas et al., 2013
ERR234247	2.2.1	Beijing	Comas et al., 2013
ERR234122	2.2.1	Beijing	Comas et al., 2013
SRR022870	2.2.1	Beijing	Comas et al., 2013
ERR234102	2.2.1	Beijing	Comas et al., 2013
ERR234130	2.2.1.1	Beijing	Comas et al., 2013
ERR234140	2.2.1.1	Beijing	Comas et al., 2013
ERR234256	2.2.1.1	Beijing	Comas et al., 2013
ERR234263	2.2.1.2	Beijing	Comas et al., 2013
ERR233359	2.2.2	Beijing	Comas et al., 2013
ERR234211	2.2.2	Beijing	Comas et al., 2013

SRR022872	2.2.2	Beijing	Comas et al., 2013
ERR234099	3	Delhi-CAS	Comas et al., 2013
ERR234181	3	Delhi-CAS	Comas et al., 2013
ERR234188	3	Delhi-CAS	Comas et al., 2013
ERR233387	3	Delhi-CAS	Comas et al., 2013
SRR022874	3	Delhi-CAS	Comas et al., 2013
ERR234111	3	Delhi-CAS	Comas et al., 2013
ERR234273	3	Delhi-CAS	Comas et al., 2013
ERR234109	3	Delhi-CAS	Comas et al., 2013
ERR234268	3	Delhi-CAS	Comas et al., 2013
ERR233385	3	Delhi-CAS	Comas et al., 2013
SRR006917	3.1.1	Delhi-CAS	Comas et al., 2013
ERX005980	3.1.1	Delhi-CAS	Comas et al., 2013
ERR234167	3.1.1	Delhi-CAS	Comas et al., 2013
ERR234166	3.1.1	Delhi-CAS	Comas et al., 2013
ERR234162	3.1.1	Delhi-CAS	Comas et al., 2013
ERR234192	3.1.2.1	Delhi-CAS	Comas et al., 2013
ERR234198	4.1	Euro-American	Comas et al., 2013
ERR234201	4.1	Euro-American	Comas et al., 2013
ERR234223	4.1.1.1	X-type	Comas et al., 2013
ERR233390	4.1.1.1	X-type	Comas et al., 2013
SRR057510	4.1.1.2	X-type	Comas et al., 2013
SRR057659	4.1.1.2	X-type	Comas et al., 2013
ERR234203	4.1.1.3	X-type	Comas et al., 2013
SRR026446	4.1.1.3	X-type	Comas et al., 2013
SRR023480	4.1.1.3	X-type	Comas et al., 2013
ERR234227	4.1.1.3	X-type	Comas et al., 2013
ERR234259	4.1.2.1	Haarlem	Comas et al., 2013
ERR234222	4.1.2.1	Haarlem	Comas et al., 2013
ERR234170	4.1.2.1	Haarlem	Comas et al., 2013
ERR233379	4.1.2.1	Haarlem	Comas et al., 2013
ERR233350	4.2.2	Euro-American	Comas et al., 2013
ERR234220	4.3.3	LAM	Comas et al., 2013
ERR234251	4.3.3	LAM	Comas et al., 2013
ERR234258	4.3.3	LAM	Comas et al., 2013
ERR234265	4.3.3	LAM	Comas et al., 2013
ERR234267	4.3.4.2	LAM	Comas et al., 2013
SRR029156	4.3.4.2	LAM	Comas et al., 2013
SRR023455	4.3.4.2	LAM	Comas et al., 2013
ERR234161	4.3.4.2.1	LAM	Comas et al., 2013
ERR233382	4.4.1.2	Euro-American	Comas et al., 2013
ERR234183	4.4.2	Euro-American	Comas et al., 2013
ERR234152	4.4.2	Euro-American	Comas et al., 2013
ERR234231	4.5	Euro-American	Comas et al., 2013
ERR234243	4.5	Euro-American	Comas et al., 2013
ERR234244	4.5	Euro-American	Comas et al., 2013
ERR234229	4.5	Euro-American	Comas et al., 2013
ERR233348	4.6	Euro-American	Comas et al., 2013
ERR234173	4.6.1.1	Uganda	Comas et al., 2013
SRR006918	4.6.1.1	Uganda	Comas et al., 2013
ERR234172	4.6.1.1	Uganda	Comas et al., 2013
ERR234171	4.6.1.2	Uganda	Comas et al., 2013
ERR234163	4.6.1.2	Uganda	Comas et al., 2013
SRR022877	4.6.2.2	Cameroon	Comas et al., 2013
ERR234200	4.6.2.2	Cameroon	Comas et al., 2013

ERR234187	4.6.2.2	Cameroon	Comas et al., 2013
ERR234228	4.7	mainly T	Comas et al., 2013
ERR234225	4.7	mainly T	Comas et al., 2013
ERR234230	4.8	mainly T	Comas et al., 2013
ERR233389	4.8	mainly T	Comas et al., 2013
ERR233369	4.8	mainly T	Comas et al., 2013
ERS457299	4.9	H37Rv-like	Feuerriegel et al., 2014
ERR1023299	4.9	H37Rv-like	Heyckendorf et al., 2018
ERR1023350	4.9	H37Rv-like	Heyckendorf et al., 2018
