# Supplementary material for: Phylogenetically Informative Mutations in Drug Resistance Genes of Human-Infecting Mycobacterium bovis
Source: Transbound Emerg Dis. 2024 Sep 26;2024:5578214. doi: 10.1155/2024/5578214 (PMC12017247; doi:10.1155/2024/5578214)
Supplement: Supporting Information S2 — A catalogue of 40 phylogenetically informative mutations in 53 drug resistance genes. [file 5578214.f2.pdf]

53 genes implicated in drug resistance				
**Gene name**	**Type**	***M. tuberculosis* H37Rv**	***M. bovis* AF2122/97**	**Associated drug resistance**
gyrB_upstream	intergenic	-	-	moxifloxacin, gatifloxacin, ofloxacin, levofloxacin
gyrB	CDS	*Rv0005*	*Mb0005*	moxifloxacin, gatifloxacin, ofloxacin, levofloxacin
gyrA	CDS	*Rv0006*	*Mb0006*	moxifloxacin, gatifloxacin, ofloxacin, levofloxacin
fgd1	CDS	*Rv0407*	*Mb0415*	delamanid, PA-824
mshA	CDS	*Rv0486*	*Mb0496*	isoniazid, ethionamide, prothionamide
ccsA	CDS	*Rv0529*	*Mb0542*	Amikacin, Capreomycin, Kanamycin
rpoB	CDS	*Rv0667*	*Mb0686*	rifampicin, rifabutin
rpoC	CDS	*Rv0668*	*Mb0687*	rifampicin, rifabutin
mmpL5	CDS	*Rv0676c*	*Mb0695c*	bedaquiline, clofazimine
rpsL_upstream	intergenic	-	-	streptomycin
rpsL	CDS	*Rv0682*	*Mb0701*	
rplC	CDS	*Rv0701*	*Mb0721*	linezolid
fbiC_upstream	intergenic	-	-	delamanid, PA-824
fbiC	CDS	*Rv1173*	*Mb1206*	delamanid, PA-824
Rv1258c	CDS	*Rv1258c*	*Mb1288c*	isoniazid, pyrazinamide, Streptomycin
embR	CDS	*Rv1267c*	*Mb1298c*	ethambutol
embR_upstream	intergenic	-	-	ethambutol
atpE_upstream	intergenic	-	-	bedaquiline
atpE	CDS	*Rv1305*	*Mb1337*	bedaquiline
rrs	rRNA	*Rvnr01*	-	streptomycin, amikacin, kanamycin, capreomycin
rrl	rRNA	*Rvnr02*	-	linezolid, capreomycin, clarithromycin
fabG1_upstream	intergenic	-	-	isoniazid, ethionamide, prothionamide
fabG1	CDS	*Rv1483*	*Mb1519*	isoniazid, ethionamide, prothionamide
inhA	CDS	*Rv1484*	*Mb1520*	isoniazid, ethionamide, prothionamide
rpsA_upstream	intergenic	-	-	pyrazinamide
rpsA	CDS	*Rv1630*	*Mb1656*	pyrazinamide
tlyA	CDS	*Rv1694*	*Mb1720*	capreomycin
ndh	CDS	*Rv1854c*	*Mb1885c*	isoniazid, ethionamide, prothionamide, clofazimine, thioridazine
ndh_upstream	intergenic	-	-	isoniazid, ethionamide, prothionamide, clofazimine, thioridazine
katG	CDS	*Rv1908c*	*Mb1943c*	isoniazid
furA	CDS	*Rv1909c*	*Mb1944c*	isoniazid
Rv1979c	CDS	*Rv1979c*	*Mb2001c*	bedaquiline, clofazimine
Rv1979c_upstream	intergenic	-	-	bedaquiline, clofazimine
pncA	CDS	*Rv2043c*	*Mb2069c*	pyrazinamide
kasA	CDS	*Rv2245*	*Mb2269*	isoniazid
eis	CDS	*Rv2416c*	*Mb2439c*	kanamycin
eis_upstream	intergenic	-	-	kanamycin
ahpC_upstream	intergenic	-	-	isoniazid
ahpC	CDS	*Rv2428*	*Mb2454*	isoniazid
folC	CDS	*Rv2447c*	*Mb2474c*	PAS
pepQ	CDS	*Rv2535c*	*Mb2564c*	bedaquiline, clofazimine
Rv2752c	CDS	*Rv2752c*	*Mb2773c*	ethambutol, isoniazid, Moxifloxacin, Rifampicin, Levofloxacin
thyX	CDS	*Rv2754c*	*Mb2775c*	PAS
thyX_upstream	intergenic	-	-	PAS
thyA	CDS	*Rv2764c*	*Mb2786c*	PAS
ald_upstream	intergenic	-	-	cycloserine
ald	CDS	*Rv2780*	*Mb2802, Mb2803*	cycloserine
fbiD	CDS	*Rv2983*	*Mb3007*	delamanid, PA-824
Rv3083	CDS	*Rv3083*	*Mb3110*	ethionamide
whiB7	CDS	*Rv3197A*	*Mb3221c*	streptomycin, kanamycin, clarithromycin, PAS
whiB7_upstream	intergenic	-	-	streptomycin, kanamycin, clarithromycin, PAS
Rv3236c	CDS	*Rv3236c*	*Mb3264c*	pyrazinamide
fbiA_upstream	intergenic	-	-	delamanid, PA-824
fbiA	CDS	*Rv3261*	*Mb3289*	delamanid, PA-823
fbiB	CDS	*Rv3262*	*Mb3290*	delamanid, PA-824
alr	CDS	*Rv3423c*	*Mb3457c*	cycloserine
alr_upstream	intergenic	-	-	cycloserine
rpoA	CDS	*Rv3457c*	*Mb3486c*	rifampicin, rifabutin
rpoA_upstream	intergenic	-	-	rifampicin, rifabutin
clpC1	CDS	*Rv3596c*	*Mb3627c*	Pyrazinamide
panD	CDS	*Rv3601c*	*Mb3631c*	pyrazinamide
embC	CDS	*Rv3793*	*Mb3822*	ethambutol
embA	CDS	*Rv3794*	*Mb3823*	ethambutol
embB	CDS	*Rv3795*	*Mb3824*	ethambutol
aftB	CDS	*Rv3805c*	*Mb3835c*	ethambutol
aftB_upstream	intergenic	-	-	ethambutol
ubiA	CDS	*Rv3806c*	*Mb3836c*	ethambutol
ethA	CDS	*Rv3854c*	*Mb3884c*	ethionamide, prothionamide, thioacetazone
ethR	CDS	*Rv3855*	*Mb3885*	ethionamide, prothionamide, thioacetazone
whiB6	CDS	*Rv3862c*	*Mb3892c*	Amikacin, Capreomycin, Kanamycin
gid	CDS	*Rv3919c*	*Mb3950c*	streptomycin
gid_upstream	intergenic	-	-	streptomycin

A catalogue of 40 phylogenetically informative mutations in 53 drug resistance genes												
**Position**	**Gene name**	***M. tuberculosis* H37Rv**	***M. bovis* AF2122/97**	**Variant Type**	**Change**	**Associated drug resistance**	***M. bovis***	***M. tuberculosis***				**Comment**
								**L1**	**L2**	**L3**	**L4**	
5752	*gyrB*	*Rv0005*	*Mb0005*	synonymous_variant	V171V (gtg/gtA)	moxifloxacin, gatifloxacin, ofloxacin, levofloxacin	+	−	−	−	−	
6406	*gyrB*	*Rv0006*	*Mb0005*	synonymous_variant	N389N (aac/aaT)	moxifloxacin, gatifloxacin, ofloxacin, levofloxacin	+	−	−	−	−	
6446	*gyrB*	*Rv0005*	*Mb0005*	missense_variant	A403S (gcg/Tcg)	moxifloxacin, gatifloxacin, ofloxacin, levofloxacin	+	−	−	−	−	
7362	*gyrA*	*Rv0006*	*Mb0006*	missense_variant	E21Q (gag/Cag)	moxifloxacin, gatifloxacin, ofloxacin, levofloxacin	+	+	+	+	±	absent in L4.9
7585	*gyrA*	*Rv0006*	*Mb0006*	missense_variant	S95T (agc/aCc)	moxifloxacin, gatifloxacin, ofloxacin, levofloxacin	+	+	+	+	±	absent in 4.7, 4.8, 4.9
8285	*gyrA*	*Rv0006*	*Mb0006*	synonymous_variant	I328I (atc/atT)	moxifloxacin, gatifloxacin, ofloxacin, levofloxacin	+	−	−	−	−	
9143	*gyrA*	*Rv0006*	*Mb0006*	synonymous_variant	I614I (att/atC)	moxifloxacin, gatifloxacin, ofloxacin, levofloxacin	+	+	−	−	−	
9304	*gyrA*	*Rv0006*	*Mb0006*	missense_variant	G668D (ggc/gAc)	moxifloxacin, gatifloxacin, ofloxacin, levofloxacin	+	+	+	+	±	absent in 4.7, 4.8, 4.9
491742	*fgd1*	*Rv0407*	*Mb0415*	synonymous_variant	F320F (ttt/ttC)	delamanid, PA-824	+	+	+	+	−	
763031	*rpoB*	*Rv0667*	*Mb0686*	upstream_gene_variant	A1075A (gct/gcC)	rifampicin, rifabutin	+	+	+	+	−	
775639	*mmpL5*	*Rv0676c*	*Mb0695c*	missense_variant	I948V (att/Gtt)	bedaquiline, clofazimine	+	+	+	+	±	absent in L4.9
776100	*mmpL5*	*Rv0676c*	*Mb0695c*	missense_variant	T794I (acc/aTc)	bedaquiline, clofazimine	+	+	+	+	−	
781395	*rpsL_upstream*	-	-	upstream_gene_variant	c.-165T>C	streptomycin	+	+	+	+	±	absent in L4.9
1302899	*fbiC_upstream*	-	-	upstream_gene_variant	c.-32A>G	delamanid, PA-824	+	−	−	−	−	
1834859	*rpsA*	*Rv1630*	*Mb1656*	missense_variant	A440T (gcg/Acg)	pyrazinamide	+	−	−	−	−	
1917972	*tlyA*	*Rv1694*	*Mb1720*	synonymous_variant	L11L (cta/ctG)	capreomycin	+	+	+	+	±	absent in L4.9
2103173	*ndh_upstream*	-	-	upstream_gene_variant	c.-132delG	isoniazid, ethionamide, prothionamide, clofazimine, thioridazine	+	−	−	−	−	
2154724	*katG*	*Rv1908c*	*Mb1943c*	missense_variant	R463L (cgg/cTg)	isoniazid	+	+	+	+	−	
2155503	*katG*	*Rv1908c*	*Mb1943c*	synonymous_variant	T203T (acc/acT)	isoniazid	+	−	−	−	−	
2156025	*katG*	*Rv1908c*	*Mb1943c*	synonymous_variant	P29P (ccc/ccA)	isoniazid	+	−	−	−	−	
2222308	*Rv1979c*	*Rv1979c*	*Mb2001c*	missense_variant	D286G (gac/gGc)	bedaquiline, clofazimine	+	+	−	−	−	
2223293	*Rv1979c_upstream*	-	-	upstream_gene_variant	c.-129A>G	bedaquiline, clofazimine	+	+	+	+	±	absent in L4.9
2518132	*kasA*	*Rv2245*	*Mb2269*	synonymous_variant	T6T (acc/acT)	isoniazid	+	+	−	−	−	
3086788	*ald_upstream*	-	-	upstream_gene_variant	c.-32T>C	cycloserine	+	+	+	+	±	absent in L4.5, 4.6, 4.7, 4.8, 4.9
3087084	*ald*	*Rv2780*	*Mb2802, Mb2803*	frameshift_variant	c.266delA p.Gln89fs	cycloserine	+	−	−	−	−	
3448783	*Rv3083*	*Rv3083*	*Mb3110*	missense_variant	V94I (gtc/Atc)	ethionamide	+	−	−	−	−	
4038403	*clpC1*	*Rv3596c*	*Mb3627c*	synonymous_variant	L768L(ttg/Ctg)	Pyrazinamide	+	−	−	−	−	
4240671	*embC*	*Rv3793*	*Mb3822*	missense_variant	T270I (acc/aTc)	ethambutol	+	+	−	−	−	
4242643	*embC*	*Rv3794*	*Mb3822*	synonymous_variant	R927R (cgc/cgT)	ethambutol	+	+	+	+	±	absent in L4.9
4242970	*embC*	*Rv3794*	*Mb3822*	upstream_gene_variant	T1036T (acc/acT)	ethambutol	+	−	−	−	−	
4244220	*embA*	*Rv3794*	*Mb3822*	synonymous_variant	L330L (ctg/Ttg)	ethambutol	+	−	−	−	−	
4246551	*embB*	*Rv3795*	*Mb3824*	missense_variant	N13S (aat/aGt)	ethambutol	+	−	−	−	−	
4246864	*embB*	*Rv3795*	*Mb3824*	synonymous_variant	V117V (gtc/gtT)	ethambutol	+	−	−	−	−	
4247646	*embB*	*Rv3795*	*Mb3824*	missense_variant	E378A (gag/gCg)	ethambutol	+	+	−	−	−	
4267858	*aftB*	*Rv3805c*	*Mb3835c*	missense_variant	I327V (att/Gtt)	ethambutol	+	−	−	−	−	
4269351	*ubiA*	*Rv3806c*	*Mb3836c*	synonymous_variant	A161A (gcc/gcT)	ethambutol	+	−	−	−	−	
4269387	*ubiA*	*Rv3806c*	*Mb3836c*	missense_variant	E149D (gaa/gaC)	ethambutol	+	+	−	−	−	
4269606	*ubiA*	*Rv3806c*	*Mb3836c*	synonymous_variant	R76R (cgt/cgC)	ethambutol	+	+	−	−	−	
4338595	*whiB6*	*Rv3862c*	*Mb3892c*	upstream_gene_variant	c.-75delG	Amikacin, Capreomycin, Kanamycin	+	+	+	+	±	absent in L4.9
4407588	*gid*	*Rv3919c*	*Mb3950c*	synonymous_variant	A205A (gca/gcG)	streptomycin	+	+	+	+	−	
